# Development and internal validation of a prediction model for new-onset macular oedema following vitrectomy for diabetic retinopathy: a single-centre retrospective analysis

**DOI:** 10.3389/fopht.2026.1932517

**Published:** 2026-10-29

**Authors:** Jiayu Ma, Jing Wang, Yiwei Wang, Chenyu Yang, Jiahao Li, Yingjun Li

**Affiliations:** 1 Department of Ophthalmology, Fuyang People’s Hospital Affiliated to Anhui Medical University, Fuyang, Anhui, China; 2 Department of Ophthalmology, Fuyang Hospital Affiliated to Bengbu Medical University (Fuyang People’s Hospital), Fuyang, Anhui, China

**Keywords:** diabetic retinopathy, macular oedema, nomogram, prediction model, SHAP, vitrectomy

## Abstract

**Objective:**

To develop and internally validate a compact model for estimating the 1-year risk of new-onset postoperative macular oedema in patients with diabetic retinopathy undergoing vitrectomy.

**Methods:**

This single-centre retrospective cohort included 650 recorded patients treated from January 2023 to April 2025. The primary analysis comprised 607 patients with ascertainable 1-year outcomes, with one study eye per patient; 43 patients lost to follow-up were assessed separately. New-onset macular oedema required new intraretinal cystoid spaces and/or subretinal fluid on optical coherence tomography during postoperative days 30–365, together with central macular thickness ≥300 μm or an increase ≥30 μm from baseline. The five-predictor logistic model used pre-incision measurements and was fitted in all 607 patients. Internal validation used 1, 000 bootstrap resamples; a 32-candidate, 10-fold cross-validated least absolute shrinkage and selection operator (LASSO) analysis with selection-inclusive bootstrap validation assessed the stability of the separate LASSO procedure.

**Results:**

New-onset postoperative macular oedema occurred in 207 of 607 patients (34.1%). The apparent area under the receiver operating characteristic curve (AUC) was 0.823 and the optimism-corrected AUC was 0.818. The corresponding Brier scores were 0.159 and 0.163; the corrected calibration intercept and slope were −0.011 and 0.969. At an illustrative probability threshold of 0.30, 40 of 323 patients (12.4%) in the lower-risk group and 167 of 284 (58.8%) in the higher-risk group developed the outcome. Selection-inclusive validation of the LASSO sensitivity model yielded a corrected AUC of 0.805. Systolic pressure was not statistically significant in the five-predictor model (P = 0.329).

**Conclusion:**

The five-variable model showed useful discrimination and acceptable bootstrap-corrected calibration in this single-centre cohort. It should be regarded as an internally validated risk-stratification model that requires external and prospective validation before clinical implementation.

## 1 Introduction

Diabetic retinopathy remains a common microvascular complication of diabetes, and recent national estimates show that the burden of vision-threatening disease is still substantial (1). In vitreoretinal practice, the clinical question is not limited to the presence of retinopathy. For patients who have already reached the stage of diabetic vitrectomy, postoperative central visual recovery becomes a more direct concern. Macular oedema is one of the main reasons why anatomical success after surgery may not translate into stable visual improvement.

Population-based work from northeast China reported that diabetic macular oedema and clinically significant macular oedema were associated with systemic factors such as longer diabetes duration, higher HbA1c, systolic blood pressure and renal-related indices (2). An OCT-based study from Turkey reached a similar conclusion from another clinical setting: diabetic macular oedema does not behave as a purely ocular abnormality; metabolic and vascular status are also relevant (3). These findings are consistent with routine surgical experience, in which postoperative oedema is shaped by both ocular conditions and the systemic background of the patient.

Blood pressure deserves particular attention. In type 2 diabetes, systolic and diastolic blood pressure have been reported as independent risk factors for diabetic retinopathy after adjustment for glycaemic status and disease duration (4). Low-grade inflammation is another plausible pathway. A cohort study in Chinese patients found that a higher neutrophil-to-lymphocyte ratio predicted incident diabetic retinopathy (5). Evidence from NHANES in the United States also supports an association between NLR and diabetic retinopathy, indicating that NLR may serve as a convenient inflammatory marker in clinical datasets (6).

Post-vitrectomy diabetic macular oedema has recently been described more directly. Zhou and colleagues reported that new postoperative oedema was common after vitrectomy for proliferative diabetic retinopathy, especially in eyes with thicker preoperative central retinal thickness (7). Vitrectomy in diabetic macular oedema has also been examined with swept-source optical coherence tomography angiography, showing that anatomical and vascular parameters can change after surgery and may relate to final visual function (8). These data point to a practical gap: clinicians collect many baseline measurements before surgery, but few tools combine them into an individual estimate of postoperative macular oedema risk.

Earlier work from the Diabetic Retinopathy Clinical Research Network showed that vitrectomy for diabetic macular oedema with vitreomacular traction reduced retinal thickening in many eyes, whereas visual gains were less uniform (9). A subsequent analysis found that preoperative visual acuity and retinal thickness carried information about postoperative visual and anatomical outcomes (10). These studies support the view that a model for postoperative macular oedema should include both systemic and ocular measurements.

For severe proliferative diabetic vitreoretinopathy, individualized management has become increasingly important because surgical indication, macular status, metabolic control and postoperative complications vary widely across patients (11). Large real-world data also show that some patients with proliferative diabetic retinopathy progress to pars plana vitrectomy despite prior retinal treatment (12). The present study therefore developed and internally validated a model for new-onset macular oedema during postoperative days 30–365 using measurements obtained before incision. The analysis emphasizes complete model specification, bootstrap internal validation and transparent reporting of model limitations.

## 2 Materials and methods

### 2.1 General information

This single-centre retrospective cohort comprised patients with diabetic retinopathy who underwent vitrectomy at Fuyang People’s Hospital between 1 January 2023 and 30 April 2025. The recorded surgical cohort contained 650 patients. The 1-year outcome could be ascertained for 607; the remaining 43 were lost before outcome ascertainment. Each patient contributed one study eye. Among 71 patients with two eligible eyes, the first eligible eye operated during the study period was selected and the fellow eye excluded. Risk was estimated immediately before incision using only measurements available by that time.

The study was conducted in accordance with the Declaration of Helsinki and was approved by the Ethics Committee of Fuyang People’s Hospital. Because this retrospective study used de-identified clinical data, the ethics committee waived the requirement for written informed consent.

The analytic eligibility criteria were: (1) diabetic retinopathy diagnosed from the history of diabetes and characteristic findings on dilated fundus examination and/or retinal imaging, in accordance with the Diabetic Retinopathy Preferred Practice Pattern (13); (2) vitrectomy at the study centre during the inclusion period; (3) age ≥18 years; (4) preoperative systemic, laboratory and ophthalmic measurements available within the specified windows; and (5) postoperative assessments sufficient to determine incident oedema on days 30–365.

Patients with active macular oedema on preoperative optical coherence tomography were not eligible for an incident-oedema analysis; a history of resolved oedema was permitted and was recorded separately. Patients were excluded from the primary analysis when postoperative macular status could not be ascertained, a primary-model predictor or the outcome was missing, or follow-up was lost before 1-year ascertainment. Cachexia, active malignancy, severe hepatic or renal dysfunction, uncontrolled systemic infection and other severe disorders that precluded assessment were clinical exclusion criteria. The number screened before entry into the 650-patient recorded cohort was unavailable.

### 2.2 Study design and preoperative assessment

Preoperative measurements were abstracted from the medical record before any intraoperative intervention. Systolic pressure and laboratory variables were obtained within 7 days before surgery; decimal best-corrected visual acuity and baseline spectral-domain optical coherence tomography, including central macular thickness, were obtained within 3 days. Blood pressure was recorded once with a calibrated automated sphygmomanometer and an appropriately sized cuff after at least 5 minutes of seated rest. Repeated measurements were not averaged. Hypertension status was recorded, but the specific antihypertensive regimen was not sufficiently standardized for analysis. Optical coherence tomography used a fovea-centred volume scan; automated segmentation was reviewed manually, and the same device platform was used for serial examinations whenever possible.

### 2.3 Outcome definition and ascertainment

The outcome was the first new episode of macular oedema detected on postoperative days 30–365. A qualifying spectral-domain optical coherence tomography (OCT) scan had to show new intraretinal cystoid spaces and/or subretinal fluid, together with central macular thickness ≥300 μm or an increase of ≥30 μm over the preoperative value. Thickness alone, including thickening attributed to traction without retinal fluid, did not satisfy the definition. Active preoperative oedema and persistent oedema were ineligible as incident outcomes; previously resolved oedema was recorded as a baseline history. Preoperative thickening without fluid did not itself constitute an incident event. The day of the first qualifying scan was assigned as the event date; recorded first events occurred on days 31–344. The model endpoint does not include oedema detected within the first 29 postoperative days.

Each of the 207 recorded events had a second qualifying OCT scan 3–14 days after the first. Two retinal specialists assessed scans independently, with a third specialist resolving disagreements; four cases underwent adjudication. The individual reading decisions were documented in source records retained at the study centre. Inter-reader agreement was not quantified in the present analysis. Patients without a qualifying event had serial OCT records through 1 year.

### 2.4 Surgical and postoperative management

All study eyes underwent pars plana vitrectomy. The surgical registry recorded retinopathy severity, the presence and macular extent of tractional retinal detachment, surgical indication, lens status, combined phacovitrectomy, 23- or 25-gauge instrumentation, endolaser and tamponade with balanced salt solution/air, expansile gas or silicone oil. Retinopathy severity, surgical indication and lens status were available before surgery but were not included in the five-predictor model. Procedural details were documented for cohort description because they were determined during surgery, after the pre-incision prediction time.

Use of anti–vascular endothelial growth factor (VEGF) therapy, corticosteroids and non-steroidal anti-inflammatory drugs after surgery was abstracted with the first treatment date. These variables describe subsequent care and were not entered as pre-incision predictors; some treatment decisions could have followed the onset of oedema. Differences in subsequent treatment between outcome groups therefore cannot establish predictive or causal treatment effects.

### 2.5 Missing data and loss to follow-up

Missingness was tabulated for the recorded fields and for the 32 candidate predictors. The five primary-model predictors, all 32 LASSO candidates and the outcome were complete for the 607 patients in the primary analysis, so no predictor imputation was performed (**Supplementary Table S1**). The 43 patients lost to follow-up retained available baseline records but had no assigned 1-year outcome. Baseline differences associated with attrition were described using standardized mean differences (**Supplementary Table S2**). Extreme scenarios assigning zero or all 43 as events bounded the event frequency among the 650 recorded patients without imputing patient-level outcomes. This analysis describes the possible event frequency but does not recover the missing outcomes or correct attrition bias.

### 2.6 Observation indicators

The 32 original candidate predictors were age, sex, body mass index, hypertension, coronary heart disease, transient ischaemic attack, chronic obstructive pulmonary disease, smoking, alcohol consumption, pulse, systolic and diastolic pressure, C-reactive protein, white blood cell count, neutrophil count, lymphocyte count, neutrophil-to-lymphocyte ratio, haemoglobin, platelet count, alanine aminotransferase, aspartate aminotransferase, albumin, creatinine, uric acid, total cholesterol, triglycerides, high-density lipoprotein cholesterol, glycated haemoglobin, fasting blood glucose, decimal best-corrected visual acuity, intraocular pressure and central macular thickness. Neutrophil-to-lymphocyte ratio was calculated by dividing the neutrophil count by the lymphocyte count. Additional descriptive variables included diabetes type and duration, estimated glomerular filtration rate, prior anti–vascular endothelial growth factor treatment, resolved diabetic macular oedema, diabetic retinopathy severity and operative and postoperative management characteristics.

### 2.7 Statistical treatment

Analyses were performed in R version 4.5.3 (R Foundation for Statistical Computing, Vienna, Austria). Continuous variables are summarized as medians and interquartile ranges and categorical variables as counts and percentages. Exploratory comparisons between outcome groups used the Wilcoxon rank-sum test and the chi-square or Fisher exact test, as appropriate. Tests were two-sided, with an unadjusted P<0.05 used as a descriptive significance threshold. These comparisons were not used to screen candidates for the LASSO sensitivity analysis. Standardized mean differences were used to assess baseline differences associated with loss to follow-up; the corresponding group summaries are arithmetic means.

The primary analysis re-estimated the original five-variable logistic model, comprising preoperative systolic pressure, neutrophil-to-lymphocyte ratio, glycated haemoglobin, decimal best-corrected visual acuity and central macular thickness. These variables represent blood pressure, systemic inflammation, glycaemic control, visual function and macular anatomy. All coefficients were re-estimated in all 607 patients, and the predictor set was held unchanged during 1, 000 bootstrap resamples. As a separate sensitivity analysis, all 32 candidates entered a LASSO logistic model without screening on outcome-group P values. Predictors were standardized internally, and ten-fold cross-validation used binomial deviance to select the penalty. The minimum-deviance penalty (lambda.min) and the largest penalty within one standard error of the minimum (lambda.1se) were reported. The lambda.1se model was used for sensitivity-model predictions and performance assessment. Cross-validation, penalty selection and predictor selection were repeated within each of 1, 000 bootstrap samples; selection frequencies refer to the lambda.1se rule. This analysis assesses selection stability of the LASSO procedure; it does not establish the selection rule of the original five-variable model.

The assumption of linearity on the logit scale was explored for each of the five continuous predictors using restricted cubic splines with knots at the 5th, 35th, 65th and 95th percentiles. Each predictor was replaced in turn by its spline terms while the other four retained linear terms; a likelihood-ratio test compared the spline and linear models. Variance inflation factors assessed collinearity. For fixed-model internal validation, 1, 000 patient-level samples of size 607 were drawn with replacement. Model coefficients were refitted in each sample, and AUC, Brier score, calibration intercept and calibration slope were evaluated in both that sample and the original cohort. The mean difference between bootstrap-sample and original-cohort performance was subtracted from the original apparent statistic to obtain the mean-optimism-corrected estimate. The 2.5th–97.5th percentiles of bootstrap training-sample apparent AUC and Brier score were reported as descriptive resampling ranges. They are not confidence intervals for the optimism-corrected estimates; such intervals were not calculated. This validation accounts for coefficient-fitting optimism conditional on the fixed predictor set, but not uncertainty arising from predictor selection. No external validation was performed.

Apparent decision curves (27) compared the model with enhanced surveillance for everyone or no one over threshold probabilities of 0.01–0.60. Thresholds of 0.10–0.40 were examined as an illustrative range for additional OCT surveillance or earlier review; no formal preference-elicitation or cost analysis established a clinical threshold. A round probability cut-off of 0.30 was used to illustrate monitoring stratification, with apparent sensitivity and specificity reported descriptively. It was not optimized using Youden’s index, selected as a validated balance of benefits and harms, or linked to preventive medication. For this additive logistic model, feature-level Shapley additive explanation values were computed on the log-odds scale as βj(xij−meanj); these explain predictions rather than causal effects (28). Reporting was checked against TRIPOD+AI guidance (26).

## 3 Results

### 3.1 Study cohort

Among the 650 patients in the recorded surgical cohort, 43 (6.6%) were lost before 1-year outcome ascertainment and 607 formed the primary analysis set (one eye per patient). New-onset postoperative macular oedema occurred in 207 patients (34.1%); 400 had no qualifying event during days 30–365 (**Figure 1**). Seventy-one patients had bilateral eligibility, and only the first eligible operated eye was retained. All five predictors and the outcome were complete in the primary analysis set.

**Figure 1:**
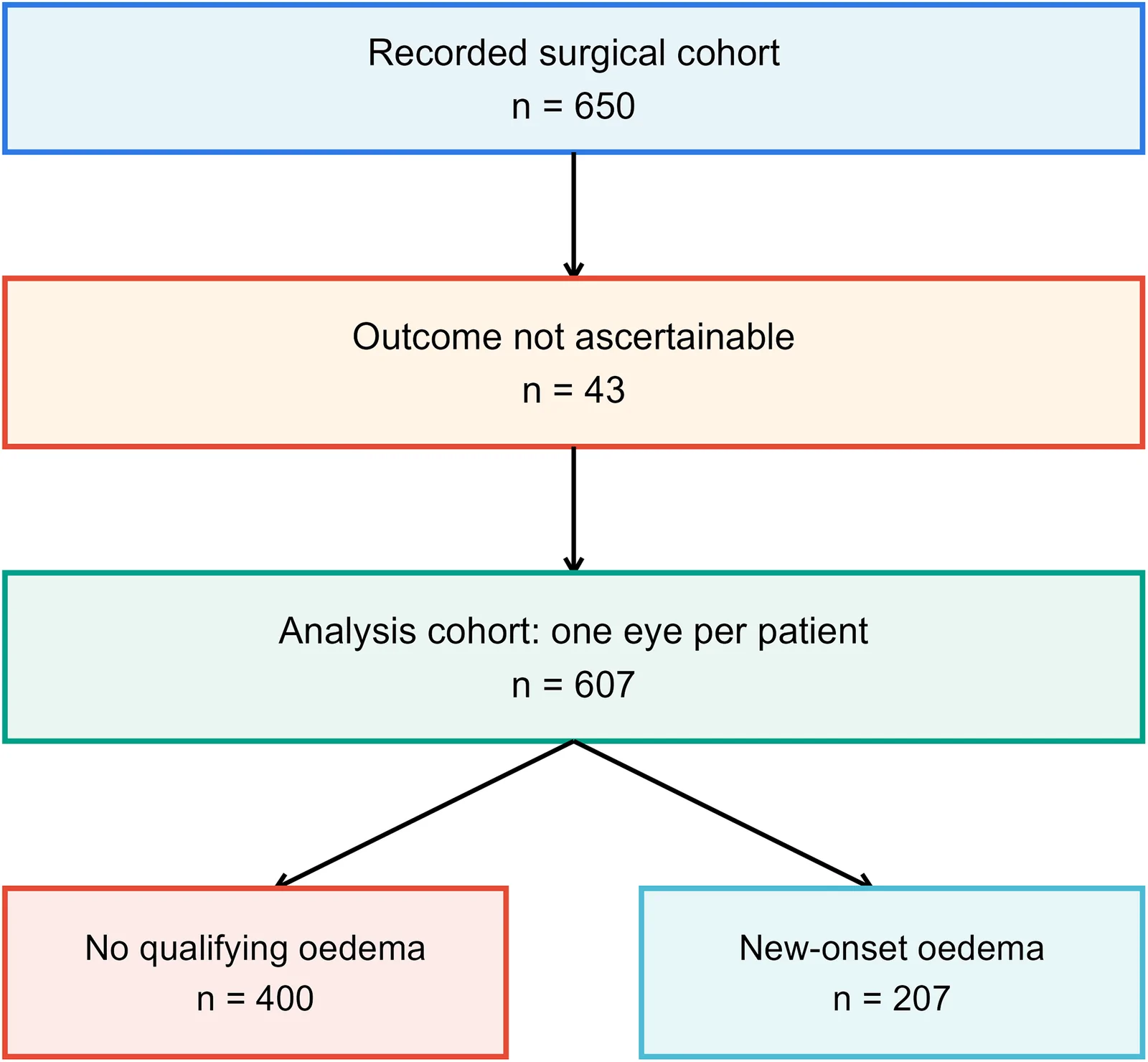
Study flow. The recorded surgical cohort contained 650 patients; 43 lacked ascertainable 1-year outcomes and 607 patients, each contributing one eye, entered the primary analysis. Of these, 207 had new-onset oedema and 400 did not. The 71 patients with bilateral eligibility contributed only their first eligible operated eye.

The largest absolute standardized mean differences between retained and lost patients were for estimated glomerular filtration rate (0.367), preoperative central macular thickness (0.252), diabetes duration (0.241) and decimal visual acuity (0.229); the other six assessed differences were ≤0.191 (**Supplementary Table S2**). If none or all 43 lost patients had developed the outcome, the event frequency among the 650 recorded patients would be 31.8% or 38.5%, respectively. These bounds do not resolve potential attrition bias.

### 3.2 Baseline, surgical and postoperative characteristics

Among the 607 analysed patients, 598 (98.5%) had type 2 diabetes and the median diabetes duration was 12 years (interquartile range, 9–15). Median glycated haemoglobin was 9.8% (8.8–10.9). Tractional retinal detachment was present in 439 eyes, including 220 with macular involvement or threat. Surgery used silicone oil in 202 eyes, expansile gas in 143 and balanced salt solution/air in 262. Postoperative anti-VEGF treatment, corticosteroids and non-steroidal anti-inflammatory drugs were recorded in 231, 121 and 320 patients, respectively. These treatments were initiated at varying times and some could have followed the qualifying event. Their distribution is descriptive and does not establish an effect on oedema risk. Baseline, ocular, surgical and subsequent-treatment characteristics are presented in **Table 1**.

**Table 1:** Baseline, ocular, surgical and postoperative characteristics.

Variable	All (n=607)	No event (n=400)	Event (n=207)	P value
Age, years	57 (51–65.5)	56 (51–62)	60 (54–69)	<0.001
Body mass index, kg/m²	24.6 (23.05–26.1)	24.45 (22.9–25.9)	24.8 (23.25–26.25)	0.074
Diabetes duration, years	12 (9–15)	11 (8–14.25)	13 (10–16)	<0.001
Estimated glomerular filtration rate, mL/min/1.73 m²	86.3 (72.5–101.7)	88.75 (73.78–102.9)	81.9 (70.1–98.1)	0.005
Systolic pressure, mmHg	131 (120–140)	128.5 (119–138)	133 (123.5–143)	<0.001
Diastolic pressure, mmHg	77 (72–83)	76 (71–82)	78 (73–84)	0.013
Neutrophil-to-lymphocyte ratio	2.48 (1.87–3.36)	2.26 (1.76–3.1)	2.82 (2.19–3.88)	<0.001
Glycated haemoglobin, %	9.8 (8.8–10.9)	9.5 (8.5–10.5)	10.3 (9.25–11.6)	<0.001
Decimal best-corrected visual acuity	0.62 (0.46–0.78)	0.69 (0.54–0.84)	0.49 (0.32–0.64)	<0.001
Central macular thickness, μm	302 (256–346)	283.5 (242–326)	341 (301–383)	<0.001
Male sex	290 (47.8%)	186 (46.5%)	104 (50.2%)	0.430
Hypertension	280 (46.1%)	174 (43.5%)	106 (51.2%)	0.085
Type 2 diabetes	598 (98.5%)	395 (98.8%)	203 (98.1%)	0.760
History of resolved diabetic macular oedema	150 (24.7%)	81 (20.2%)	69 (33.3%)	<0.001
Anti-vascular endothelial growth factor treatment within 6 months	253 (41.7%)	166 (41.5%)	87 (42.0%)	0.969
Proliferative diabetic retinopathy severity: without tractional retinal detachment	168 (27.7%)	120 (30.0%)	48 (23.2%)	0.051
Proliferative diabetic retinopathy severity: tractional retinal detachment sparing macula	219 (36.1%)	148 (37.0%)	71 (34.3%)	
Proliferative diabetic retinopathy severity: tractional retinal detachment involving/threatening macula	220 (36.2%)	132 (33.0%)	88 (42.5%)	
Macular tractional retinal detachment involvement	220 (36.2%)	132 (33.0%)	88 (42.5%)	0.026
Surgical indication: non-clearing vitreous haemorrhage	168 (27.7%)	120 (30.0%)	48 (23.2%)	0.085
Surgical indication: tractional retinal detachment	341 (56.2%)	212 (53.0%)	129 (62.3%)	
Surgical indication: combined haemorrhage and traction	98 (16.1%)	68 (17.0%)	30 (14.5%)	
Phakic lens status	340 (56.0%)	227 (56.8%)	113 (54.6%)	0.673
Combined phacovitrectomy	208 (34.3%)	132 (33.0%)	76 (36.7%)	0.410
25-gauge vitrectomy	495 (81.5%)	329 (82.2%)	166 (80.2%)	0.611
Intraoperative endolaser	553 (91.1%)	365 (91.2%)	188 (90.8%)	0.980
Tamponade: balanced salt solution/air	262 (43.2%)	172 (43.0%)	90 (43.5%)	0.444
Tamponade: expansile gas	143 (23.6%)	89 (22.2%)	54 (26.1%)	
Tamponade: silicone oil	202 (33.3%)	139 (34.8%)	63 (30.4%)	
Postoperative anti-vascular endothelial growth factor treatment	231 (38.1%)	127 (31.8%)	104 (50.2%)	<0.001
Postoperative corticosteroid treatment	121 (19.9%)	60 (15.0%)	61 (29.5%)	<0.001
Postoperative non-steroidal anti-inflammatory drug treatment	320 (52.7%)	199 (49.8%)	121 (58.5%)	0.051

Values are median (interquartile range) or n (%). P values are unadjusted exploratory univariable comparisons. Operative and postoperative variables are descriptive and were not used as pre-incision predictors. Postoperative treatments may have followed the qualifying outcome, and group differences should not be interpreted as predictive or causal treatment effects. TRD, tractional retinal detachment.

### 3.3 Candidate selection and primary model specification

In the 32-candidate LASSO sensitivity analysis, lambda.min was 0.014882 and selected 13 variables, whereas lambda.1se was 0.045447 and selected six: lymphocyte count, neutrophil-to-lymphocyte ratio, haemoglobin, glycated haemoglobin, decimal best-corrected visual acuity and central macular thickness (**Supplementary Table S1**; **Supplementary Figures S1, S2**). In 1, 000 selection-inclusive resamples, central macular thickness and visual acuity were each selected in 100%, glycated haemoglobin in 99.9%, haemoglobin in 81.2%, lymphocyte count in 74.3%, neutrophil-to-lymphocyte ratio in 63.2% and systolic pressure in 27.7%. The apparent AUC of the LASSO model was 0.820 and the selection-inclusive optimism-corrected AUC was 0.805; the corresponding Brier scores were 0.164 and 0.170. These are internal estimates, not independent validation results (**Supplementary Figure S3**).

In the primary five-predictor model, neutrophil-to-lymphocyte ratio, glycated haemoglobin and central macular thickness had positive coefficients, whereas better decimal best-corrected visual acuity had a negative coefficient. Systolic pressure was not statistically significant (coefficient 0.007664 per mmHg; odds ratio 1.008, 95% confidence interval 0.992–1.023; P = 0.329) and was not interpreted as an independent risk factor (**Table 2**). The prediction equation in original measurement units is:

**Table 2:** Five-predictor multivariable logistic regression model.

Term	Coefficient (95% CI)	OR per original unit (95% CI)	OR per SD (95% CI)	P value
Intercept	-7.935 (-10.849 to -5.022)	0.000 (0.000 to 0.007)	—	<0.001
SP	0.008 (-0.008 to 0.023)	1.008 (0.992 to 1.023)	1.109 (0.901 to 1.364)	0.329
NLR	0.287 (0.127 to 0.446)	1.332 (1.135 to 1.562)	1.470 (1.186 to 1.822)	<0.001
HbA1c	0.337 (0.189 to 0.484)	1.400 (1.208 to 1.622)	1.626 (1.315 to 2.012)	<0.001
BCVA	-2.530 (-3.529 to -1.531)	0.080 (0.029 to 0.216)	0.541 (0.425 to 0.690)	<0.001
CMT	0.011 (0.008 to 0.015)	1.012 (1.008 to 1.015)	2.087 (1.636 to 2.663)	<0.001

The prediction equation gives the original-unit coefficients at greater precision than the rounded values shown here. Standardized odds ratios were calculated as exp(β×sample standard deviation). Systolic pressure was not statistically significant and was not interpreted as an independent risk factor. CI, confidence interval; OR, odds ratio; SD, standard deviation.


logit(p)=−7.93532293+0.00766389×SP+0.28651053×NLR+0.33650951×HbA1c−2.52994910×BCVA+0.01148647×CMT

$$p=\frac{1}{1+exp\left(-logit\left(p\right)\right)}$$

SP is measured in mmHg, HbA1c in %, BCVA as decimal acuity and CMT in μm.

### 3.4 Model performance

The apparent AUC was 0.823 and the apparent Brier score was 0.159. With the five predictors held fixed in 1, 000 bootstrap resamples, mean-optimism-corrected AUC and Brier score were 0.818 and 0.163. The corresponding 2.5th–97.5th percentile ranges for bootstrap training-sample apparent AUC and Brier score were 0.790–0.857 and 0.141–0.173, respectively; these are descriptive resampling ranges rather than confidence intervals for the corrected estimates. The apparent calibration intercept and slope were approximately 0 and 1, reflecting assessment in the model-fitting data; the corresponding optimism-corrected estimates were −0.011 and 0.969 (**Table 3**; **Figures 2a, b**). Exploratory likelihood-ratio tests of nonlinearity yielded P = 0.789 for systolic pressure, 0.293 for neutrophil-to-lymphocyte ratio, 0.200 for glycated haemoglobin, 0.144 for decimal best-corrected visual acuity and 0.907 for central macular thickness. Variance inflation factors ranged from 1.076 to 1.344. These findings did not detect a departure from the linear-logit specification but do not establish linearity outside the observed predictor ranges.

**Table 3:** Bootstrap internal validation of the five-predictor model.

Metric	Apparent	Mean-optimism-corrected	Bootstrap apparent range (2.5th–97.5th percentiles)
Area under the receiver operating characteristic curve	0.823	0.818	0.790–0.857
Brier score	0.159	0.163	0.141–0.173
Calibration intercept	-0.000	-0.011	—
Calibration slope	1.000	0.969	—

Fixed-model validation used 1, 000 patient-level bootstrap resamples with the five predictors held fixed. Corrected values subtract the mean difference between bootstrap-sample apparent performance and performance in the original cohort. The ranges shown are the 2.5th–97.5th percentiles of bootstrap training-sample apparent AUC and Brier score; they are descriptive and are not confidence intervals for the corrected estimates. Confidence intervals for the corrected values were not calculated. A dash indicates that a descriptive range is not reported.

**Figure 2:**
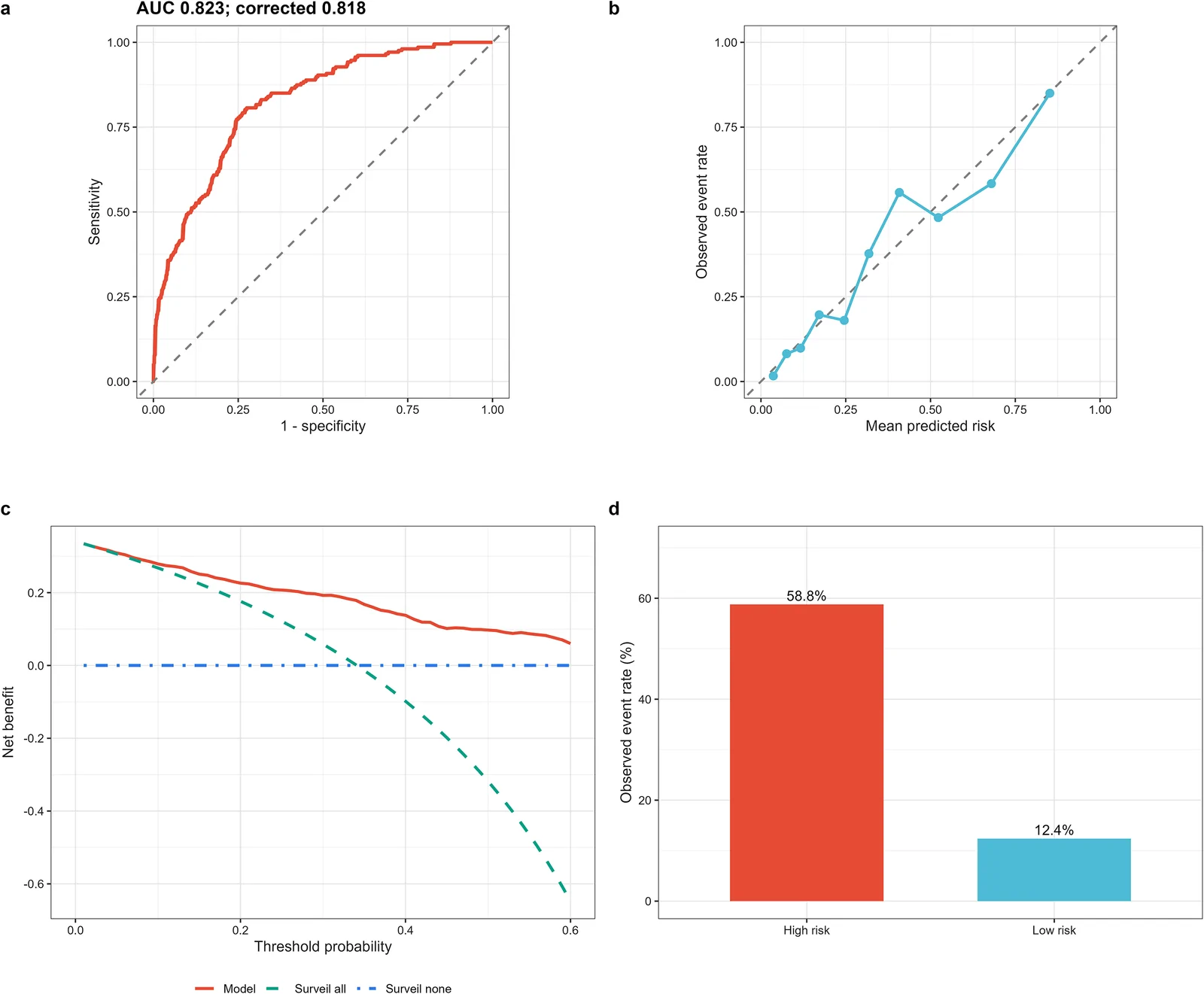
Performance of the five-predictor model in the analysis cohort. **(a)** Apparent receiver operating characteristic curve, with apparent and optimism-corrected AUC values stated above the panel. **(b)** Observed event frequency versus mean predicted risk by decile; the dashed line denotes ideal calibration. **(c)** Apparent decision curves comparing enhanced surveillance directed by the model with enhanced surveillance for everyone or no one over thresholds 0.01–0.60. **(d)** Observed event proportions below and at or above the illustrative 0.30 risk cut-off. The decision curves and risk groups are in-sample descriptions and do not establish a clinical intervention threshold.

In the apparent decision analysis, the model had greater net benefit than enhanced surveillance for everyone or no one over much of the illustrative 0.10–0.40 threshold range (**Figure 2c**). At the descriptive 0.30 cut-off, 323 patients were classified below the cut-off, including 40 events (12.4%), and 284 were classified at or above it, including 167 events (58.8%) (**Figure 2d**). Apparent sensitivity and specificity at this cut-off were 80.7% and 70.8%, respectively. These in-sample summaries illustrate risk separation; they do not establish a clinically optimal or validated intervention threshold.

### 3.5 Nomogram and feature contribution

The original-unit logistic equation was translated into a nomogram (**Figure 3**); the odds ratios per standard deviation in **Table 2** are scale conversions of the same coefficients. Exact additive-logit Shapley values ranked central macular thickness first (mean absolute contribution, 0.592 log-odds), followed by best-corrected visual acuity (0.494), glycated haemoglobin (0.397), neutrophil-to-lymphocyte ratio (0.279) and systolic pressure (0.083) (**Figure 4**). These values describe contributions to fitted predictions and do not establish causation.

**Figure 3:**
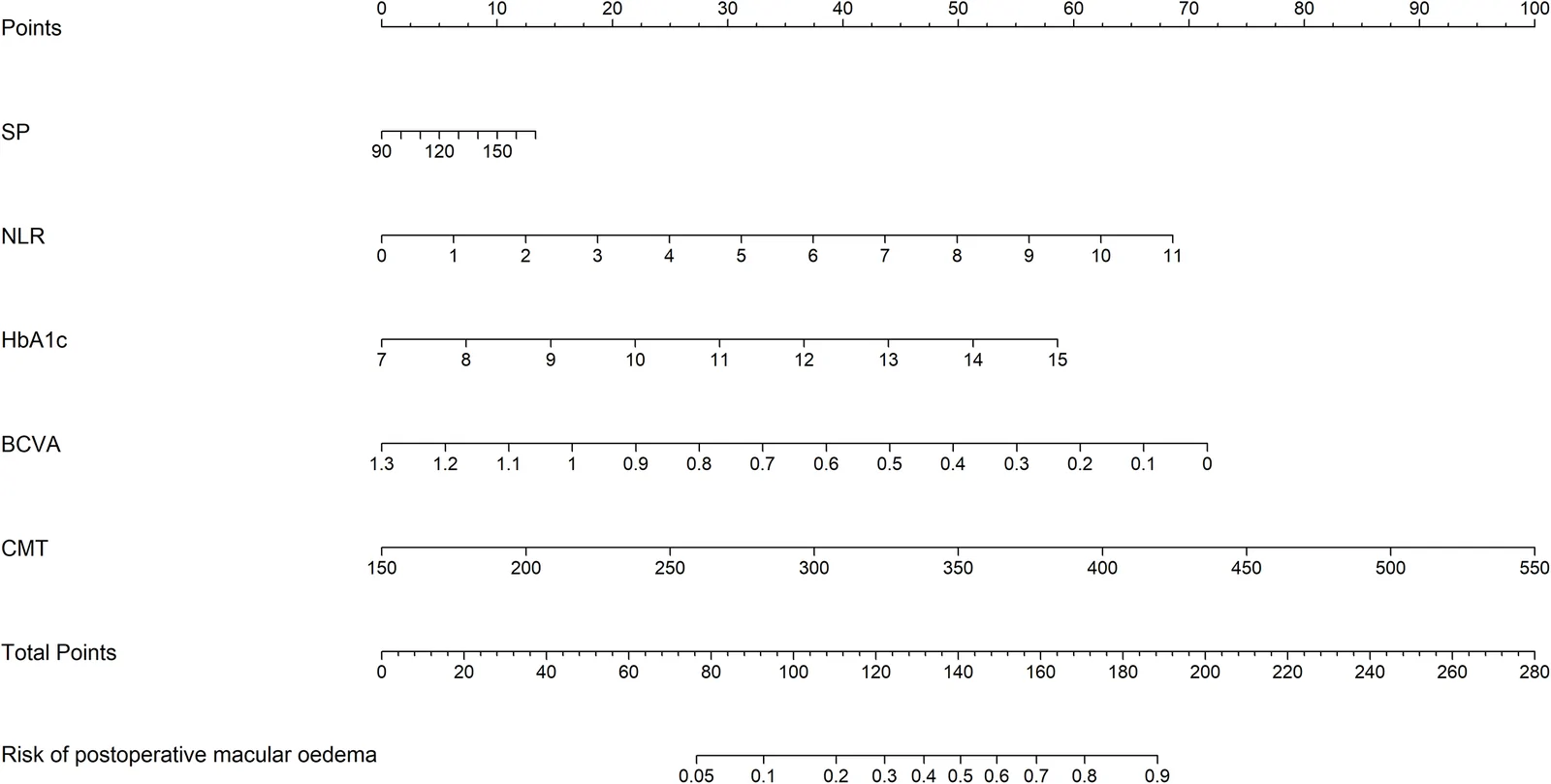
Nomogram derived from the original-unit logistic regression coefficients. SP, systolic pressure; NLR, neutrophil-to-lymphocyte ratio; HbA1c, glycated haemoglobin; BCVA, best-corrected visual acuity; CMT, central macular thickness.

**Figure 4:**
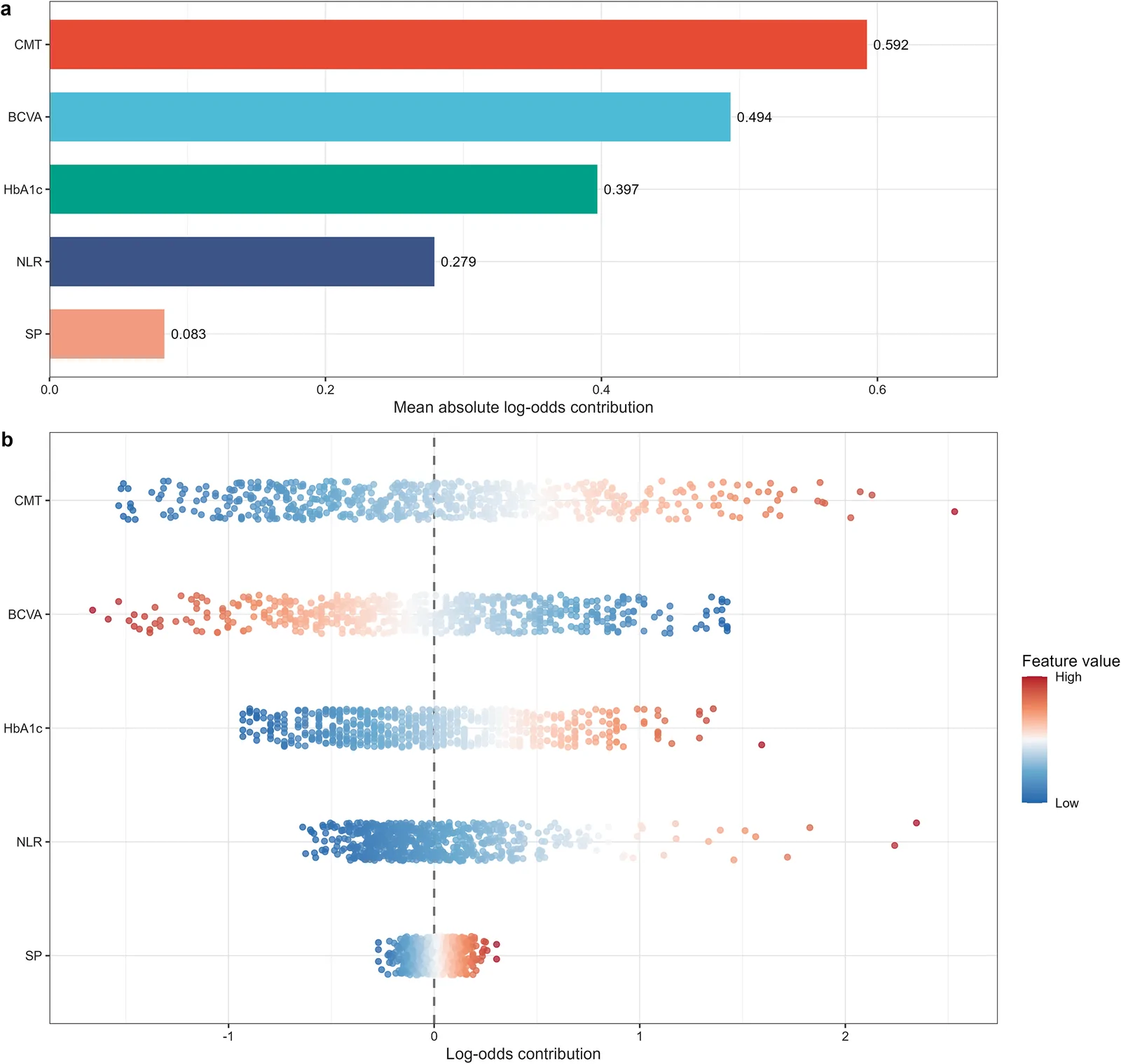
Model-based feature contribution. **(a)** Mean absolute Shapley additive explanation value on the log-odds scale. **(b)** Patient-level additive log-odds contributions centred on the analysis-set mean. These values explain model predictions and do not represent causal effects.

## 4 Discussion

This study developed a five-variable logistic model for new-onset macular oedema on postoperative days 30–365 after diabetic vitrectomy. In 607 patients, apparent AUC was 0.823 and mean-optimism-corrected AUC was 0.818. The corrected calibration slope was 0.969. These performance estimates arise from internal validation within the development cohort. The model warrants independent evaluation before clinical use.

Central macular thickness showed the largest standardized effect and the largest mean absolute Shapley contribution. Because it was measured within 3 days before surgery, it reflects preoperative macular anatomy rather than early postoperative change. A thicker macula may summarize tissue vulnerability, residual tractional effects and microvascular permeability; however, these mechanisms were not tested in this prediction study. The association is consistent with reports linking preoperative macular status and tractional severity to postoperative outcomes (14, 15).

Glycated haemoglobin and neutrophil-to-lymphocyte ratio contributed additional systemic information. The markedly elevated glycated haemoglobin distribution—median 9.8% and interquartile range 8.8%–10.9%—should be interpreted in the context of a referred severe proliferative diabetic retinopathy population rather than a general diabetes population. Systolic pressure had a small, nonsignificant coefficient and the lowest mean absolute Shapley contribution, and it should not be described as an independent risk factor in this cohort. A single standardized preoperative blood-pressure measurement may also have introduced random measurement error, and the lack of standardized antihypertensive regimen data limits further interpretation.

Better preoperative decimal best-corrected visual acuity was associated with lower predicted risk. This direction is compatible with the broader observation that retinal treatment outcomes depend on baseline functional and anatomical status (16–23). Nevertheless, the present endpoint was new-onset postoperative oedema rather than visual response to intravitreal treatment, and the coefficient should be interpreted as a predictive association within this cohort.

The LASSO sensitivity analysis selected a partly different predictor set. With the one-standard-error penalty, lymphocyte count and haemoglobin were retained, while systolic pressure was not; several candidate predictors showed variable selection across resamples. Selection-inclusive validation reduced the LASSO AUC from an apparent 0.820 to 0.805. Differences in predictor retention across modelling procedures and resamples indicate variable-selection uncertainty.

The decision curve concerns the possible value of additional OCT surveillance or earlier review. It cannot justify prophylactic anti-VEGF treatment or corticosteroids. The threshold range and the 0.30 risk-group cut-off were illustrative; neither reflects an externally established clinical preference or was tested prospectively. The separation in observed event rates therefore requires replication before it informs a monitoring protocol.

This model differs from machine-learning studies that predict response to anti–vascular endothelial growth factor treatment in diabetic macular oedema (24, 25). It predicts a postoperative complication from five routinely recorded pre-incision measurements and provides a complete equation and nomogram. The transparent logistic form is an advantage for reproducibility, but simplicity does not remove the need to test transportability across institutions, surgeons, imaging platforms and patient populations.

Reporting followed TRIPOD+AI guidance for regression and machine-learning prediction models (26). All 607 patients contributed to model estimation, with bootstrap internal validation used instead of relying on a single random split. This avoids dependence on a particular random partition and permits coefficient estimation using the full analysis cohort, but it does not test performance in an independent population. The full original-unit equation, calibration intercept and slope, and separate analyses of fixed-model fitting and LASSO selection are reported. Neither bootstrap analysis replaces external validation.

Several limitations remain. First, this retrospective single-centre analysis lacks external or prospective validation, and clinical impact was not assessed. Second, the 43 patients without ascertainable 1-year outcomes differed from those retained, most notably in renal function, central macular thickness, diabetes duration and visual acuity; the extreme event-rate scenarios cannot correct possible attrition bias. Third, although OCT scans were read independently and disagreements were adjudicated, inter-reader agreement was not quantified, so residual outcome-classification error cannot be excluded. Fourth, operative details and postoperative treatments were descriptive, and differences in surgical technique, tamponade and later treatment could affect transportability. Fifth, one preoperative blood-pressure reading and routine-record OCT measurements may contain error. Finally, bootstrap performance estimates were conditional on the five-variable specification and did not include uncertainty arising from predictor selection. The separate LASSO analysis assessed selection stability but cannot quantify selection optimism in the primary model. Confidence intervals for the optimism-corrected metrics were not calculated.

## 5 Conclusion

This five-variable model provides an internally validated estimate of incident macular oedema risk after diabetic vitrectomy. Its performance supports external and prospective assessment, but the nomogram and illustrative 0.30 cut-off should not yet guide clinical decisions.

## Data Availability

The raw data supporting the conclusions of this article will be made available by the authors, without undue reservation.
