# Supplementary material for: Development and internal validation of a prediction model for new-onset macular oedema following vitrectomy for diabetic retinopathy: a single-centre retrospective analysis
Source: Front Ophthalmol (Lausanne). 2026 Oct 29;6:1932517. doi: 10.3389/fopht.2026.1932517 (PMC13754739; doi:10.3389/fopht.2026.1932517)
Supplement: Supplementary file 1 [file DataSheet1.pdf]

Supplementary Material

Supplementary Figure S1 Ten-fold cross-validation curve for the 32-candidate LASSO sensitivity analysis. Vertical lines indicate the minimum-deviance and one-standard-error penalties.

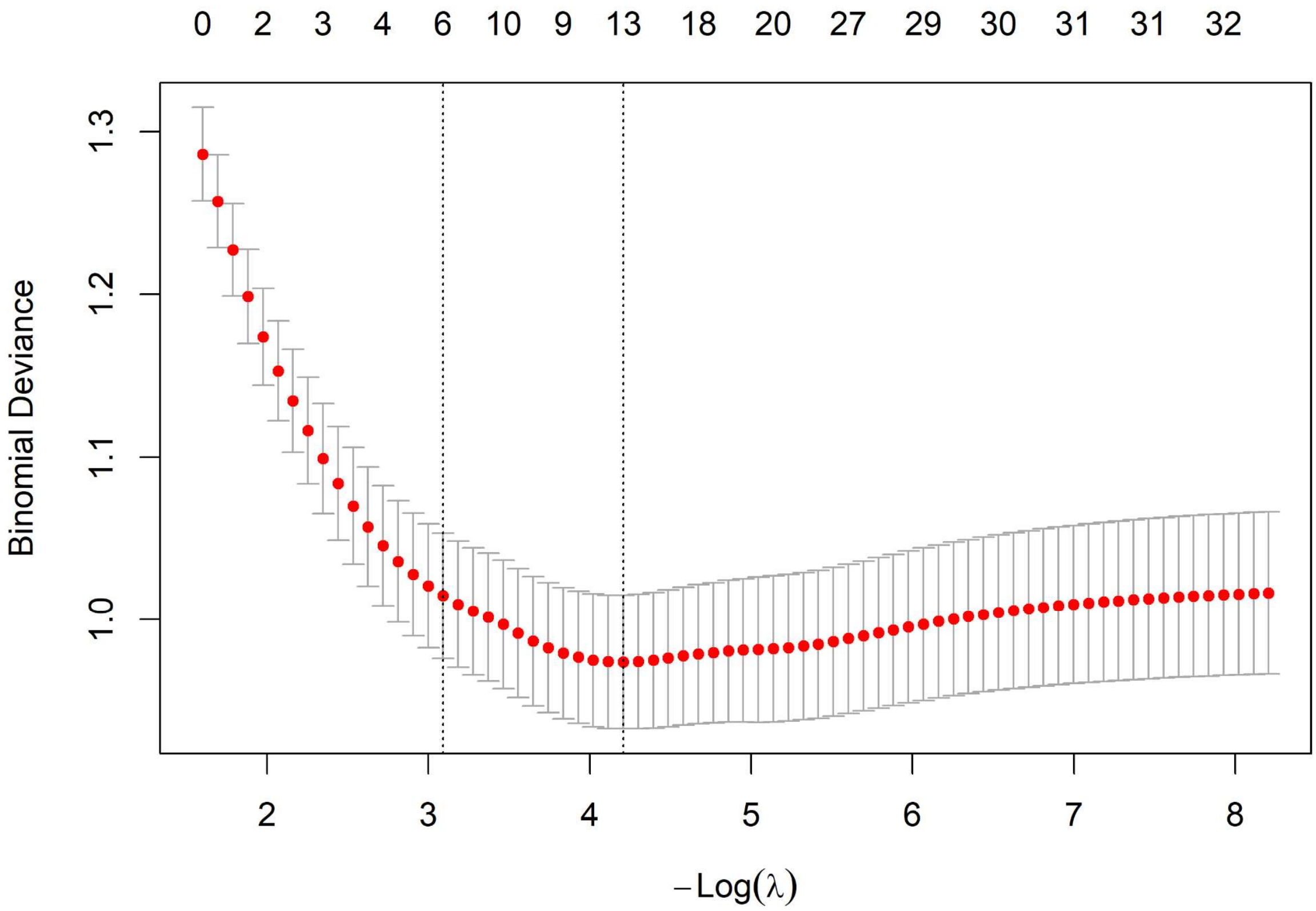

Supplementary Figure S2 Coefficient paths for the 32-candidate LASSO logistic model across penalty values.

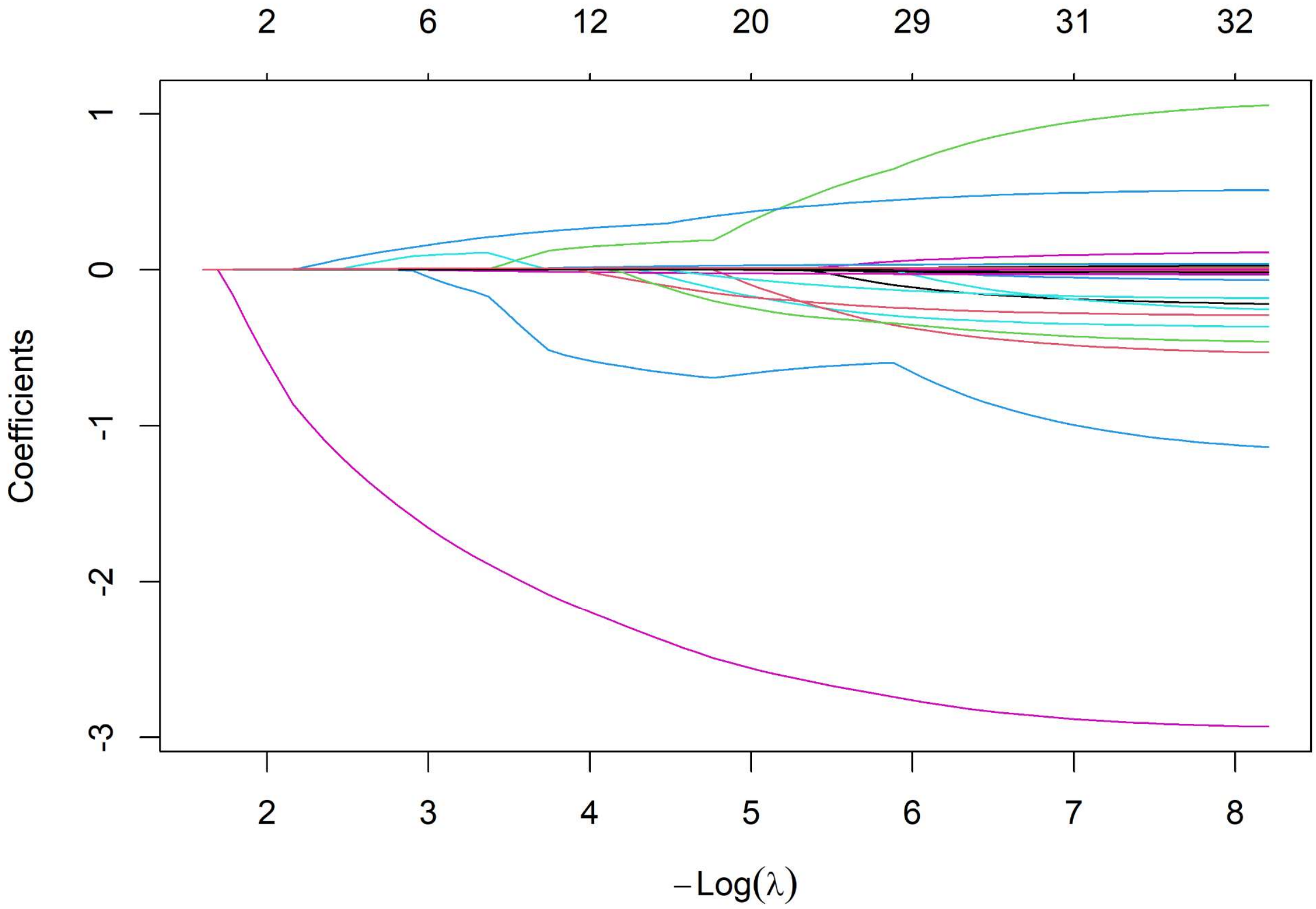

Supplementary Figure S3 Apparent AUCs of the five-predictor logistic model and the LASSO sensitivity model in the same 607-patient cohort; the chart does not show optimism-corrected estimates.

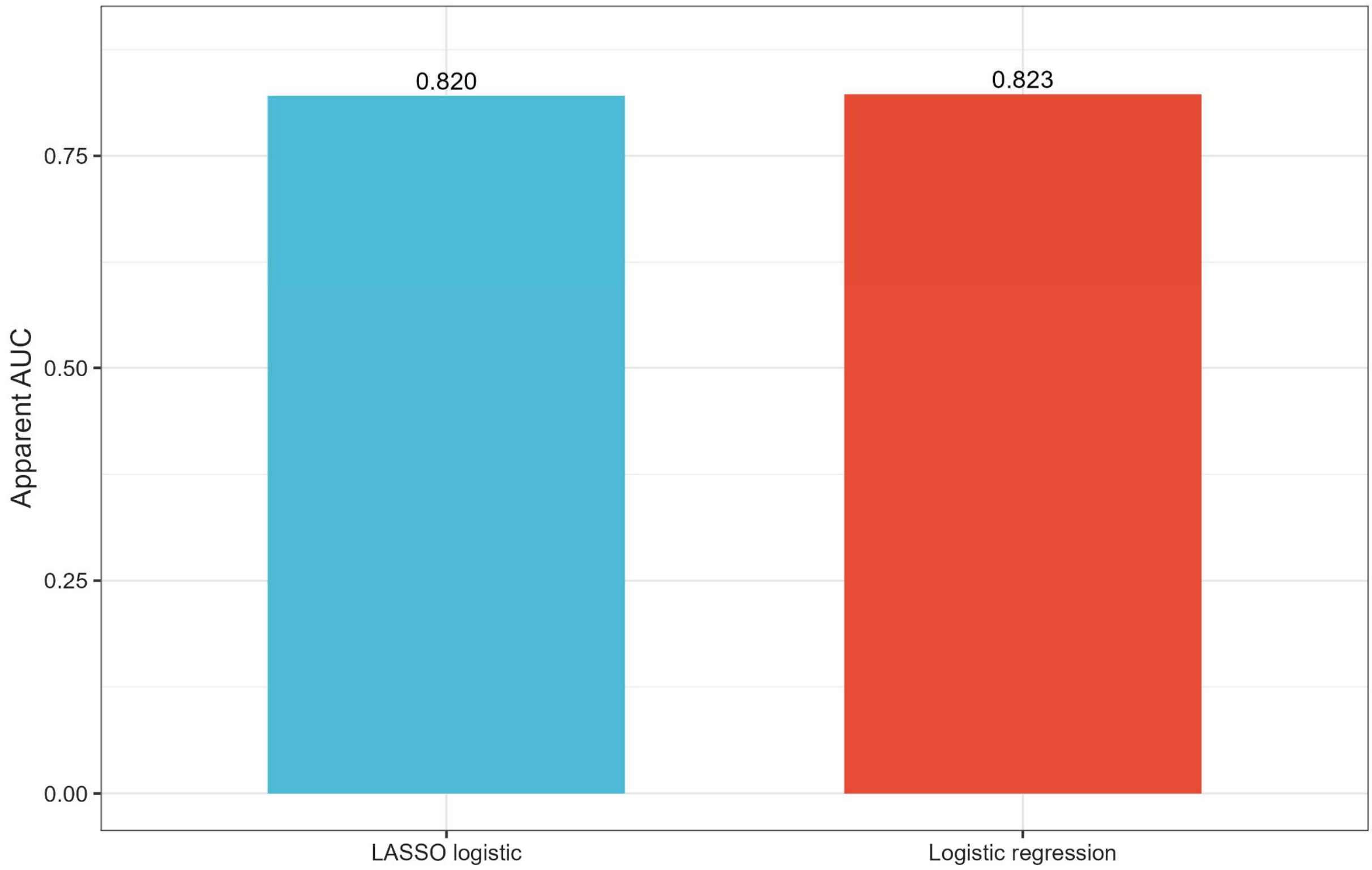

Supplementary Table S1 Missingness, LASSO selection and bootstrap selection frequency for the 32 candidate predictors

Candidate	Missing in primary analysis, n	Selected at lambda.min	Selected at lambda.1se	Bootstrap selection frequency, %
Age	0	Yes	No	52.1
Gender	0	No	No	6.3
BMI	0	No	No	12.8
Hypertension	0	No	No	8.7
CHD	0	No	No	10.7
TIA	0	No	No	14.6
COPD	0	No	No	7.1
Smoke	0	No	No	10.0
Drink	0	Yes	No	51.3
Pulse	0	Yes	No	59.1
SP	0	Yes	No	27.7
DP	0	No	No	18.1
CRP	0	No	No	8.3
WBC	0	No	No	1.9
NE	0	Yes	No	58.9
LYM	0	Yes	Yes	74.3
NLR	0	No	Yes	63.2
Hb	0	Yes	Yes	81.2
Plt	0	No	No	6.3
ALT	0	No	No	14.0
AST	0	No	No	8.8
ALB	0	No	No	13.5
Cr	0	Yes	No	24.5
UA	0	No	No	11.2
cholesterol	0	No	No	8.0
TG	0	Yes	No	24.3
HDL	0	Yes	No	16.9
HbA1c	0	Yes	Yes	99.9
FBG	0	No	No	5.4
BCVA	0	Yes	Yes	100.0

Candidate	Missing in primary analysis, n	Selected at lambda.min	Selected at lambda.1se	Bootstrap selection frequency, %
IOP	0	No	No	6.3
CMT	0	Yes	Yes	100.0

Notes: There were no missing values among the 32 candidates in the 607-patient analysis set. lambda.min=0.014882; lambda.1se=0.045447. Frequencies reflect 1,000 resamples with cross-validation and selection repeated using lambda.1se. LASSO, least absolute shrinkage and selection operator.

Supplementary Table S2 Baseline comparison of patients retained and lost to follow-up

Variable	Retained (n=607), mean	Lost (n=43), mean	SMD	Absolute SMD
Age	57.755	57.721	0.003	0.003
BMI	24.593	24.807	-0.086	0.086
SP	130.367	128.674	0.124	0.124
DP	77.072	75.558	0.191	0.191
NLR	2.762	2.708	0.039	0.039
HbA1c	9.865	9.995	-0.092	0.092
BCVA	0.623	0.680	-0.229	0.229
Preoperative CMT (μm)	304.585	287.581	0.252	0.252
Diabetes duration (years)	12.300	13.416	-0.241	0.241
eGFR	86.038	78.551	0.367	0.367

Notes: Group summaries are arithmetic means. Standardized mean differences compare the 607 retained and 43 lost patients. The unknown outcomes of the 43 lost patients were not imputed; assigning zero or all as events gives overall event frequencies of 31.8% and 38.5%, respectively. SMD, standardized mean difference.
